# Lipoprotein(a) and Atrial Fibrillation: Mechanistic Insights and Therapeutic Approaches

**DOI:** 10.7150/ijms.102301

**Published:** 2025-01-01

**Authors:** Zixi Zhang, Bo Peng, Akedanmu Nuranmubieke, Yangfan Xu, Yan Liu, Tao Tu, Qiuzhen Lin, Cancan Wang, Qiming Liu, Yichao Xiao

**Affiliations:** 1 Department of Cardiology, The Second Xiangya Hospital, Central South University, Changsha 410011, Hunan Province, People's Republic of China.; 2 Xiangya School of Medicine, Central South University, Changsha 410011, Hunan Province, People's Republic of China.; 3 Department of Endocrinology, The Second Xiangya Hospital, Central South University, Changsha 410011, Hunan Province, People's Republic of China.

**Keywords:** Lipoprotein(a), Atrial fibrillation, Cardiovascular disease, Pathogenic mechanism, Therapies

## Abstract

Elevated lipoprotein(a) [Lp(a)] levels are increasingly recognized as a significant risk factor for cardiovascular diseases and may also contribute to atrial fibrillation (AF). This review investigated the indirect mechanisms through which Lp(a) may influence AF, including proatherogenic, prothrombotic, and proinflammatory pathways. Traditional lipid-lowering therapies, such as lifestyle modifications and statins, have limited effects on Lp(a) levels. Emerging treatments, such as proprotein convertase subtilisin/kexin type 9 (PCSK9) inhibitors, lipoprotein apheresis, small interfering RNA, antisense oligonucleotides, cholesterol ester transfer protein inhibitors, and interleukin-6 receptor monoclonal antibodies, are promising alternatives. Notably, only PCSK9 inhibitors and lipoprotein apheresis have been shown to reduce both Lp(a) levels and cardiovascular events. Research indicates varying associations between Lp(a) and AF across different populations, underscoring the need for diverse, large-scale studies to elucidate these differences. Ongoing trials aim to provide clearer insights into these relationships. Addressing these gaps is essential for developing targeted therapies to manage elevated Lp(a) and mitigate the risk of AF and associated cardiovascular events.

## Introduction

**Figure 1:**
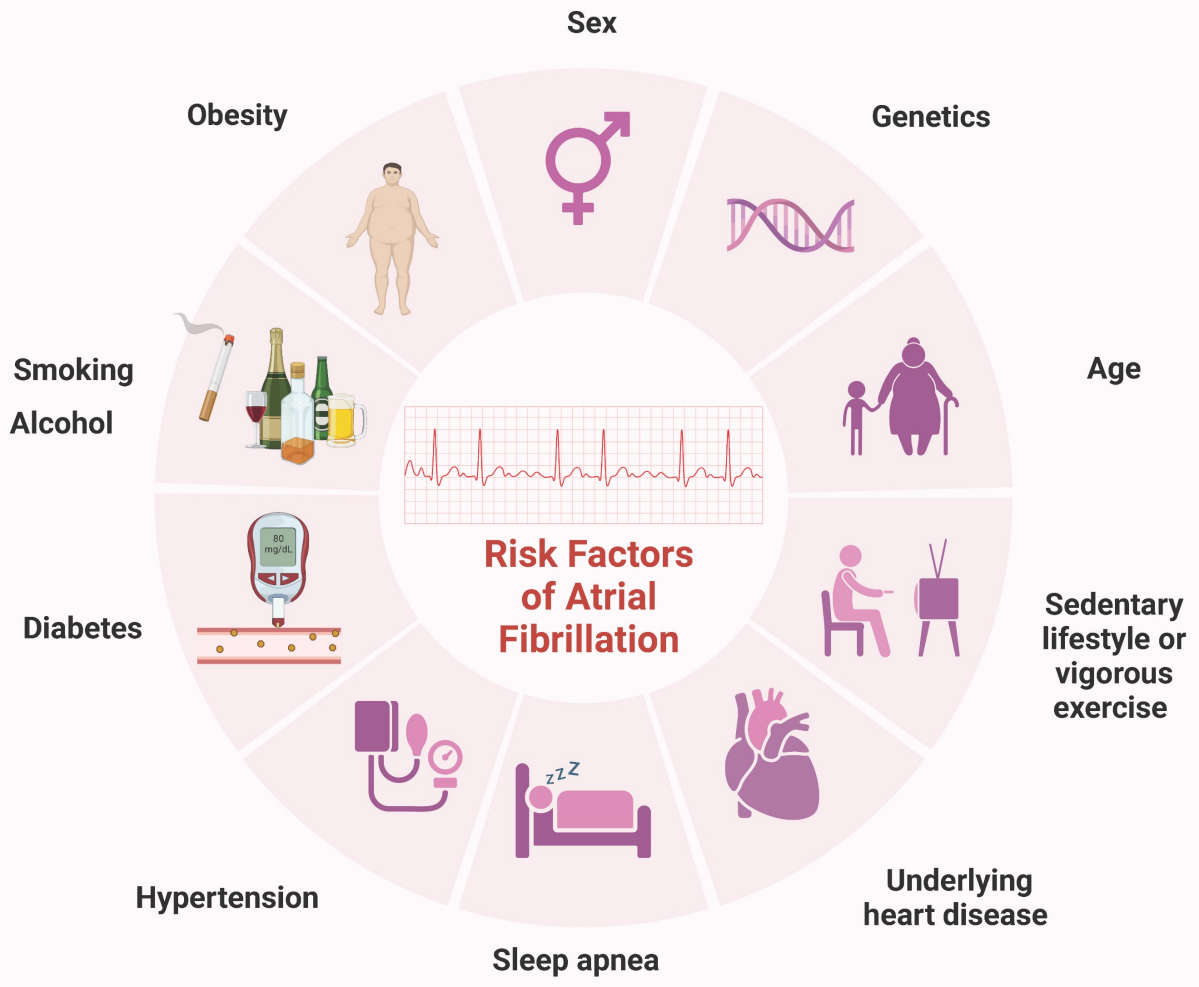
** Common risk factors for AF.** Sex, age, genetics, history of underlying heart disease, particularly congestive HF and valvular disease, hypertension, obesity, diabetes, obstructive sleep apnea, smoking, vigorous exercise, sedentary lifestyle, smoking and alcohol have all been linked to the incidence of AF. AF, atrial fibrillation; HF, heart failure.

Atrial fibrillation (AF) is the most common sustained arrhythmia in adults and represents a significant global public health challenge. In 2010, over 33 million individuals worldwide were affected by AF, with higher incidence and prevalence rates observed in developed countries 1. Projections indicate that by 2030, the European Union will have 14-17 million AF patients, with an annual diagnosis of 120000-215000 new cases 2. AF is influenced by various factors, including sex, age, genetics, hypertension, and obesity 3, 4. Other potential contributors include diabetes, smoking, obstructive sleep apnea, vigorous exercise, sedentary lifestyles, and alcohol consumption 5, 6 (Figure 1). This complex array of risk factors underscores the necessity for ongoing research to identify additional contributors and to develop novel treatment and prevention strategies.

In this context, the role of lipoprotein(a) [Lp(a)] has garnered significant attention. Lp(a) is a low-density lipoprotein cholesterol (LDL-C)-like lipid particle composed of cholesterol, cholesteryl esters, apolipoprotein B100, apolipoprotein(a) [Apo(a)], and small amounts of triglycerides and carbohydrates. It is synthesized primarily in the liver and is metabolized in the liver or kidneys 7, 8. Current studies suggest that elevated Lp(a) levels may increase the risk of cardiovascular disease (CVD), identifying Lp(a) as an independent marker of atherosclerosis 9. Notably, Lp(a) levels are genetically determined and exhibit significant variation among different ethnic groups. While some studies have linked elevated Lp(a) levels to a greater risk of AF, others have suggested that increased Lp(a) may reduce the risk of AF 10-12. The pathogenic mechanisms of Lp(a) include proatherogenic, prothrombotic, and proinflammatory processes 13, 14. However, the exact role of Lp(a) in AF risk remains unclear and may be related to these mechanisms. A deeper understanding of the relationship between Lp(a) and AF is crucial for developing novel prevention and treatment strategies.

This article reviews the mechanisms by which Lp(a) may contribute to the development of AF and its role in AF-related comorbidities. We also explore treatment strategies for reducing Lp(a) levels to clarify the connection between Lp(a) and AF, providing new insights for the management and prevention of AF.

## Conflicting evidence and racial variability

**Figure 2:**
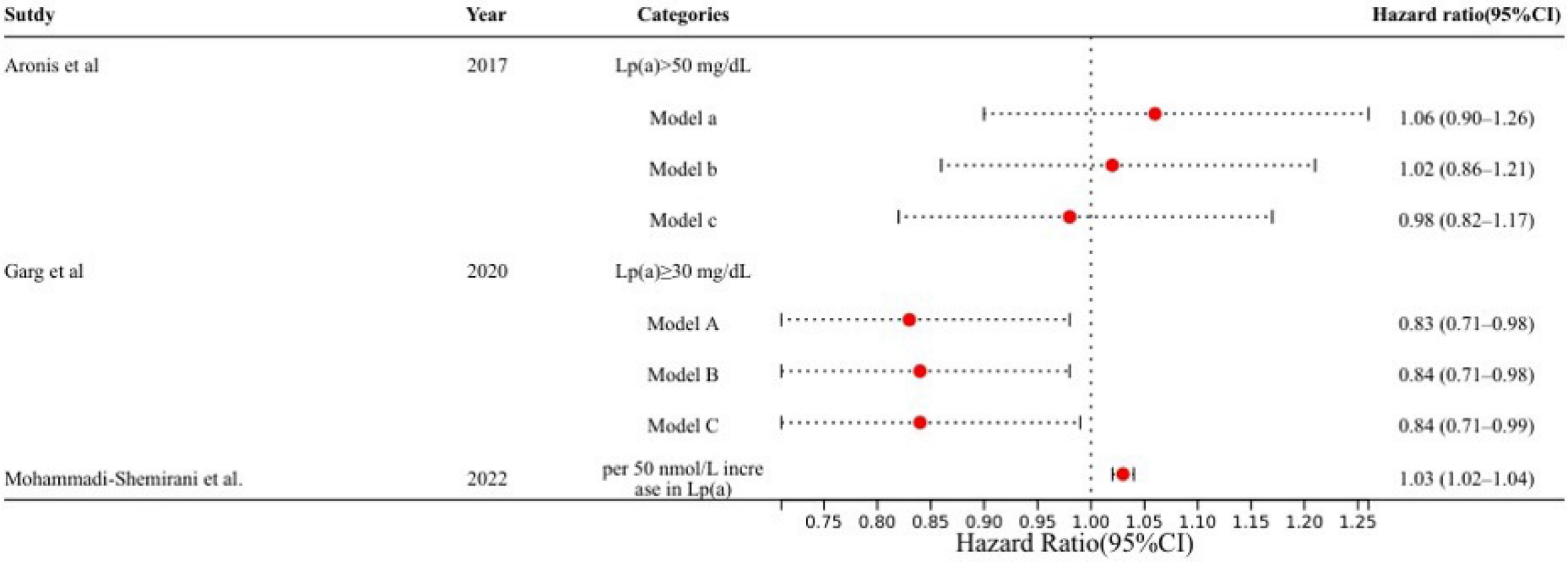
** Adjusted models examining the associations between Lp(a) levels and AF in recent studies.** Model a: Adjusted for age, sex, and ethnic groupings. Model b: Model a was extended by adding smoking status, systolic and diastolic blood pressure, hypertension therapy, heart rate, height, BMI, ECG left ventricular hypertrophy, PR interval, prevalent HF, CAD, and diabetes mellitus. Model c: Model b was further extended by adding LDL cholesterol, HDL cholesterol, triglycerides, lipid-lowering medication, and a log-transformed N-terminal pro-B-type natriuretic peptide. Model A: Adjusted for age, race, sex, education, income, and location. Model B: Model A was extended by adding height, BMI, smoking status, diabetes mellitus status, SBP, DBP, antihypertensive medication, physical activity, and alcohol intake. Model C further extended Model B by adding total cholesterol, HDL cholesterol, triglycerides, and lipid-lowering treatment. AF, atrial fibrillation; BMI, body mass index; CAD, coronary artery disease; DBP, diastolic blood pressure; ECG, electrocardiogram; HDL, high-density lipoprotein; HF, heart failure; LDL, low-density lipoprotein; Lp(a), lipoprotein(a); SBP, systolic blood pressure.

Previous studies have indicated that elevated Lp(a) levels increase the risk of CVD. Research by Satterfield *et al.* 15 revealed that elevated Lp(a) levels in European pedigrees contributed to AF, suggesting potential clinical implications. However, in 2014, Mora *et al.* 16 reported no relationship between Lp(a) and AF. This study has several limitations, including a reliance on a single baseline lipoprotein measurement, a lack of continuous electrocardiogram (ECG) monitoring, and the potential exclusion of asymptomatic AF patients. Furthermore, the study population consisted solely of women, which may introduce demographic biases. In 2017, Aronis *et al.* 12 reported that high Lp(a) levels were not associated with incident AF, but this study excluded individuals with extreme phenotypes of high Lp(a) levels (i.e., > 100 mg/dL). Moreover, the study included only white and black participants, raising concerns about the generalizability of the results to other ethnic groups. A 2020 study by Garg *et al.* 17 revealed that participants with Lp(a) levels above 30 mg/dL had a lower chance of developing AF. Conversely, Jiang *et al.* 18 demonstrated that genetically elevated Lp(a) levels increased AF risk. However, their results were only theoretically significant and did not account for multiple hypothesis testing, potentially affecting the robustness of their findings. The study by Mohammadi-Shemirani *et al.* 19 addressed a common limitation of earlier observational studies—the limited number of participants—by utilizing the UK Biobank, thereby increasing the number of AF cases by up to 20-fold. These findings, which are based on epidemiological and genomic studies, suggest that Lp(a) might be a causal mediator of AF (Figure 2). Higher Lp(a) levels in the UK Biobank were linked with an increased risk of AF incidence. This study also revealed that Lp(a) has a specific residual effect on AF, independent of its association with ischemic heart disease or aortic valve stenosis.

**Figure 3:**
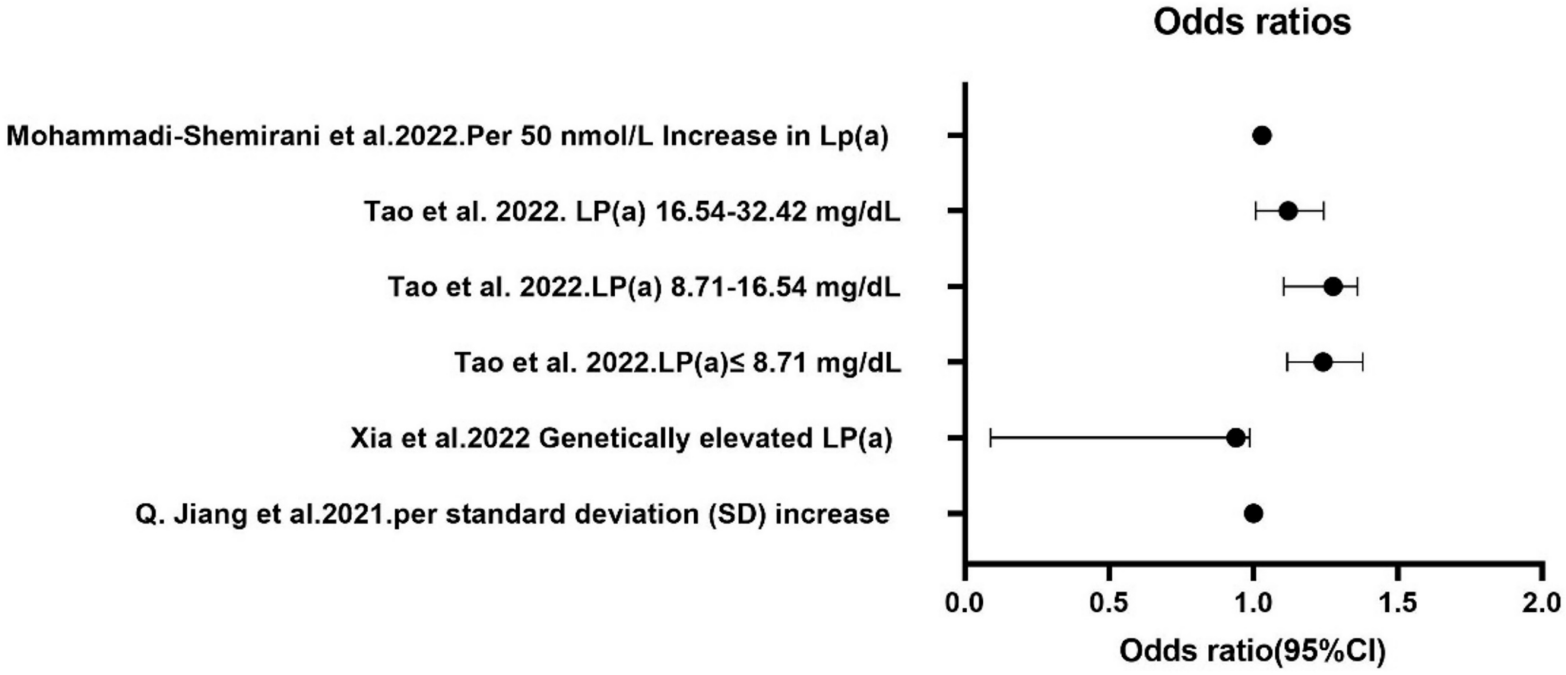
** Comparison of Lp(a) levels and AF risk in European and Asian populations.** The results from several independent studies investigating the relationship between Lp(a) levels and the risk of AF. The findings illustrate notable disparities in AF risk associated with Lp(a) levels between European and Asian populations. AF, atrial fibrillation; Lp(a), lipoprotein(a).

A common limitation of the above studies is that they included only white and black populations, leaving it unclear whether these findings apply to other ethnicities, such as Han Chinese. Lp(a) levels vary significantly across different races, and whether these findings can be generalized to other populations remains uncertain. This area requires further exploration. Additionally, the specific subcomponents of Lp(a) that affect AF and their mechanisms of action remain unanswered questions. Xia *et al.* 11 proposed that hereditary Lp(a) levels were not linked with AF among the Han Chinese population. However, a study by Tao *et al.* 20 revealed that low circulating Lp(a) levels were associated with AF, particularly in Han Chinese women, suggesting that Lp(a) might help stratify AF risk in females. The varying results from these studies may be due to differences in Lp(a) levels between races (Figure 3).

While evidence suggests a positive correlation between high Lp(a) levels and AF, the relationship is complex and influenced by racial and demographic factors. Future research should focus on identifying specific targets of Lp(a) for treating and preventing AF. Large-scale randomized controlled trials (RCTs) are needed to confirm whether these findings apply to East Asian populations and to clarify the mechanisms by which Lp(a) influences AF risk.

## Pathogenic mechanisms linking Lp(a) to AF

**Figure 4:**
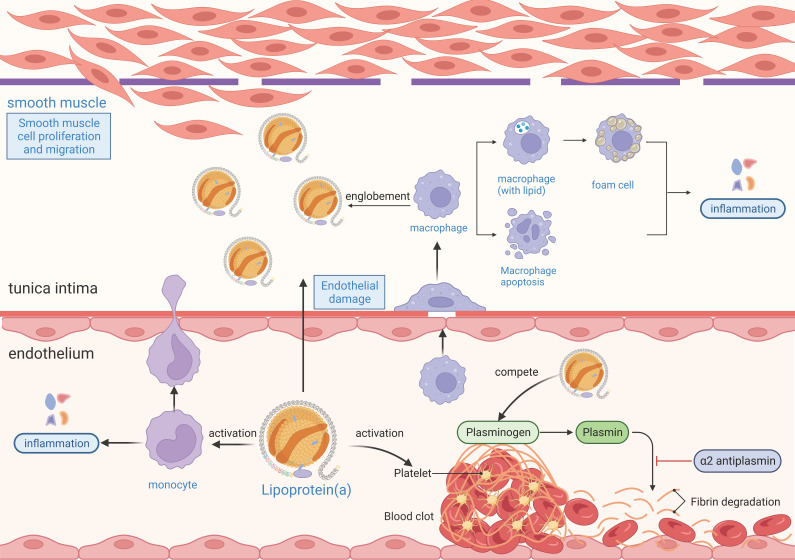
** The proatherogenic, prothrombotic, and proinflammatory mechanisms of Lp(a).** Lp(a) enters the endothelium and is readily oxidized, damaging endothelial cells and smooth muscle cells. The damaged endothelium secretes cytokines or growth factors to induce inflammatory cells such as macrophages and monocytes to enter the endothelium to phagocytose lipids and form foam cells. The damaged endothelium secretes growth factors that activate smooth muscle cells to proliferate and migrate, producing an extracellular matrix that increases the thickness and stiffness of the intima. The foam cells in the intima become necrotic and disintegrate to form atheromatous plaques. The inflammatory response occurs throughout the process of atherosclerosis, and OxPLs in Lp(a) are potent proinflammatory mediators. Lp(a) promotes thrombosis by exacerbating endothelial damage and activating platelet-associated responses, and high concentrations of Lp(a) may inhibit fibrinolytic responses by inhibiting fibrinogen activation. Lp(a), lipoprotein(a); OxPL, oxidized phospholipid.

Understanding the pathogenic mechanisms linking Lp(a) to AF is critical for identifying potential therapeutic targets. The relationship between Lp(a) function and AF pathogenesis can be explored from three main perspectives: proatherogenic, prothrombotic, and proinflammatory mechanisms (Figure 4).

### Proatherogenic mechanisms

Lp(a) plays a significant role in the development of atherosclerosis. Studies indicate that Lp(a) bound to oxidized phospholipids (OxPLs) is more readily taken up by macrophages, increasing their atherogenic potential 21. Elevated levels of Lp(a) can directly damage the vascular wall and exacerbate atherosclerosis through inflammatory responses. Moreover, the OxPL component of Lp(a) activates endothelial cells and promotes monocyte migration across the endothelium, worsening atherosclerosis 22, 23. Atherosclerosis can mediate the occurrence of AF. The results from an observational and Mendelian randomization study demonstrated that for each 50 nmol/L (23 mg/dL) genetically predicted increase in Lp(a), the risk of AF increased by 3% [95% confidence interval (CI): 1.02-1.05]. Mendelian randomization via two independent genome-wide association studies (GWASs) provided evidence of a positive causal relationship between Lp(a) levels and AF 19. Additionally, a meta-analysis of Mendelian randomization data demonstrated that elevated Lp(a) levels were associated with an increased risk of AF [odds ratio (OR): 1.024, 95% CI: 1.007-1.042, I² = 87.72%; *P* < 0.001], with notable variations across different ethnic groups. In European populations, higher Lp(a) levels were linked to a significantly increased risk of AF (OR: 1.023, 95% CI: 1.007-1.040; *P* < 0.001). However, in Chinese populations, elevated Lp(a) levels are associated with a comparatively lower risk of AF (OR: 0.940, 95% CI: 0.893-0.990) 24. These findings suggest that Lp(a) is a potential causal mediator of AF development, with effects that are partly independent of atherosclerotic cardiovascular disease (ASCVD). Atherosclerosis affects atrial structure and electrical function through various mechanisms, including inflammation, myocardial ischemia, atrial infarction, myocardial cell death, atrial fibrosis, and coronary artery narrowing, all of which can contribute to AF 25. Among these factors, endothelial dysfunction is considered a key element in the development of AF in patients with atherosclerosis. During AF episodes, rapid atrial pacing can decrease shear stress on blood vessels, reduce nitric oxide production by the endothelium, and increase oxidative stress markers, potentially creating a vicious cycle 26. Given the multifaceted role of Lp(a) in promoting atherosclerosis and its downstream impact on AF development, targeting endothelial dysfunction has emerged as a promising therapeutic strategy. Research has shown that statins can improve endothelial dysfunction in both peripheral and coronary arteries, reduce the risk of AF, and lower the recurrence rate of AF after catheter ablation or dual-chamber pacemaker implantation 27-29. These findings underscore the importance of comprehensive management approaches to address both the atherosclerotic burden and its arrhythmogenic complications.

The role of Lp(a) in promoting atherosclerosis and influencing AF is complex and multifaceted. Lp(a) can impact atrial structure and function both directly and indirectly. Future research should further explore the specific mechanisms by which Lp(a) contributes to atherosclerosis and its associated AF risk and assess whether reducing Lp(a) levels could effectively decrease the incidence and progression of AF.

### Prothrombotic mechanisms

Lp(a) is closely associated with an increased risk of thrombosis in patients with AF. One of the key components of Lp(a) is Apo(a), which shares structural similarities with plasminogen but has different physiological functions. Apo(a) inhibits tissue plasminogen activator, preventing the conversion of plasminogen to plasmin 30, thereby reducing fibrinolysis and impairing the body's ability to break down clots, promoting thrombus formation. Moreover, Lp(a) enhances coagulation by increasing platelet reactivity, upregulating tissue factor expression, and inhibiting the tissue factor pathway 31, 32. Although the interaction between Lp(a) and platelets has been studied, the exact receptor involved remains unclear. Current evidence on this interaction is conflicting, with some studies suggesting that Lp(a) may have both activating and inhibitory effects depending on the activation pathway. For example, Lp(a) reportedly enhances platelet activation via thrombin-related activating hexapeptides, although it does not directly interact with thrombin or adenosine diphosphate 33. Conversely, other studies have indicated that Lp(a) inhibits platelet activation induced by collagen or thrombin 34. Despite these discrepancies, there is broad consensus that Lp(a) significantly impairs fibrinolysis by competing with plasminogen and tissue plasminogen activator for binding sites on the platelet surface, thereby obstructing thrombus resolution 35. This prothrombotic activity contributes to a hypercoagulable state and is associated with systemic inflammation and atrial fibrosis, both of which are linked to adverse outcomes in AF patients 36. Elevated Lp(a) levels are particularly concerning because they correlate with the progression of atrial fibrosis and the disruption of atrial structure and electrical activity, increasing the risk of clot formation 37. Additionally, Lp(a) may exacerbate inflammatory responses in the atrial endocardium 38, further increasing thrombotic risk.

There is increasing evidence that AF is associated with a prothrombotic state characterized by various hematological abnormalities, including elevated Lp(a) levels 39, 40. Igarashi *et al.* 41 reported a correlation between thrombi in the left atrium of elderly patients with chronic AF and elevated Lp(a) levels, although this study had a small sample size. Another study on nonvalvular AF revealed that high Lp(a) levels were linked to thrombotic events in individuals with low CHA_2_DS_2_-VASc scores 42. A cross-sectional investigation revealed that elevated Lp(a) levels in patients with nonvalvular AF increased the likelihood of ischemic stroke and thrombosis 43. These findings collectively suggest a significant association between elevated Lp(a) levels and increased thrombotic risk in patients with AF.

Given the role of Lp(a) in promoting coagulation and inhibiting fibrinolysis in AF patients, targeting Lp(a) with specific treatments could be clinically significant 44. Recent studies have shown that proprotein convertase subtilisin/kexin type 9 (PCSK9) inhibitors can significantly lower Lp(a) levels 45, 46, potentially offering benefits in thrombus prevention for AF patients. Further research is needed to evaluate the effectiveness of these therapeutic strategies in reducing thrombotic events in AF patients and to determine how these strategies can be integrated into comprehensive AF management plans.

### Proinflammatory mechanisms

Recent evidence highlights the proinflammatory effects of Lp(a), which are driven primarily by its OxPL content 47. OxPLs associated with Lp(a) are potent proinflammatory mediators that contribute to cardiovascular inflammation and play significant roles in endothelial dysfunction, vascular inflammation, and oxidative stress 48. Current research indicates that AF mechanisms are intricately linked to atrial electrical and structural remodeling, processes heavily influenced by inflammation 49-51. Inflammatory mediators modulate ion channels and calcium homeostasis, disrupt cardiac action potential duration, and promote aberrant calcium handling in cardiomyocytes. Such dysregulation fosters atrial ectopic activity and reentry circuits, which are critical in AF initiation and perpetuation. Concurrently, inflammation activates fibroblasts, driving excessive collagen deposition and atrial fibrosis while also inducing cardiomyocyte apoptosis, further contributing to atrial structural remodeling 52.

Inflammation is closely associated with both the prevalence and progression of AF. Mediators of the inflammatory response can alter the electrophysiological and structural substrates of the atria, thereby increasing susceptibility to AF. A cross-sectional study demonstrated that elevated levels of interleukin-6 (IL-6) were associated with the development of both paroxysmal and chronic AF 53. Additionally, a study by Jia *et al.* 54 revealed that AF patients with higher IL-6 levels had a significantly increased risk of stroke [hazard ratio (HR): 3.81, 95% CI: 1.11-13.05; *P* = 0.033) and all-cause mortality (HR: 3.11, 95% CI: 1.25-7.72; *P* = 0.015). Furthermore, risk factors for AF, such as obesity, diabetes, hypertension, and metabolic syndrome, are also associated with inflammatory states 55. These findings indicate that inflammation plays a crucial role in the onset and progression of AF. Notably, OxPLs carried by Lp(a) can amplify these inflammatory pathways by activating Toll-like receptors and downstream nuclear factor-kappa B signaling, both of which have been implicated in atrial fibrosis and oxidative stress 56. This dual proinflammatory and pro-oxidative action creates a pathological environment conducive to AF. Oxidative stress, exacerbated by OxPLs, further initiates the remodeling process. Excess reactive oxygen species (ROS) promote the oxidation of calcium-handling proteins, such as ryanodine receptors, impairing their function and contributing to intracellular calcium overload—a hallmark of AF 57. ROS also facilitate the activation of matrix metalloproteinases, enzymes that degrade extracellular matrix components, disrupting atrial structural integrity and promoting fibrosis 58.

Given the overlap between OxPL-mediated inflammation, oxidative stress, and AF pathogenesis, targeting Lp(a) and its associated OxPLs represents a promising therapeutic strategy. Anti-inflammatory approaches, such as interleukin-1 blockers and Toll-like receptor inhibitors, may mitigate atrial remodeling and slow fibrosis progression, thereby preserving atrial structure and function. Preclinical studies have shown that strategies that reduce ROS levels can improve calcium homeostasis and prevent electrical remodeling, highlighting the potential of antioxidative therapies in AF management 59-61.

Future investigations should focus on the direct modulation of Lp(a)-OxPL pathways in AF through clinical trials, exploring agents such as PCSK9 inhibitors and antisense oligonucleotides to reduce Lp(a) levels. Additionally, therapies targeting oxidative stress and inflammation, potentially in combination, could offer synergistic benefits by addressing both structural and electrical remodeling, thereby reducing the AF burden and improving patient outcomes.

## Elevated Lp(a) and cardiovascular risk

### Association of elevated Lp(a) with AF and coronary artery disease

**Figure 5:**
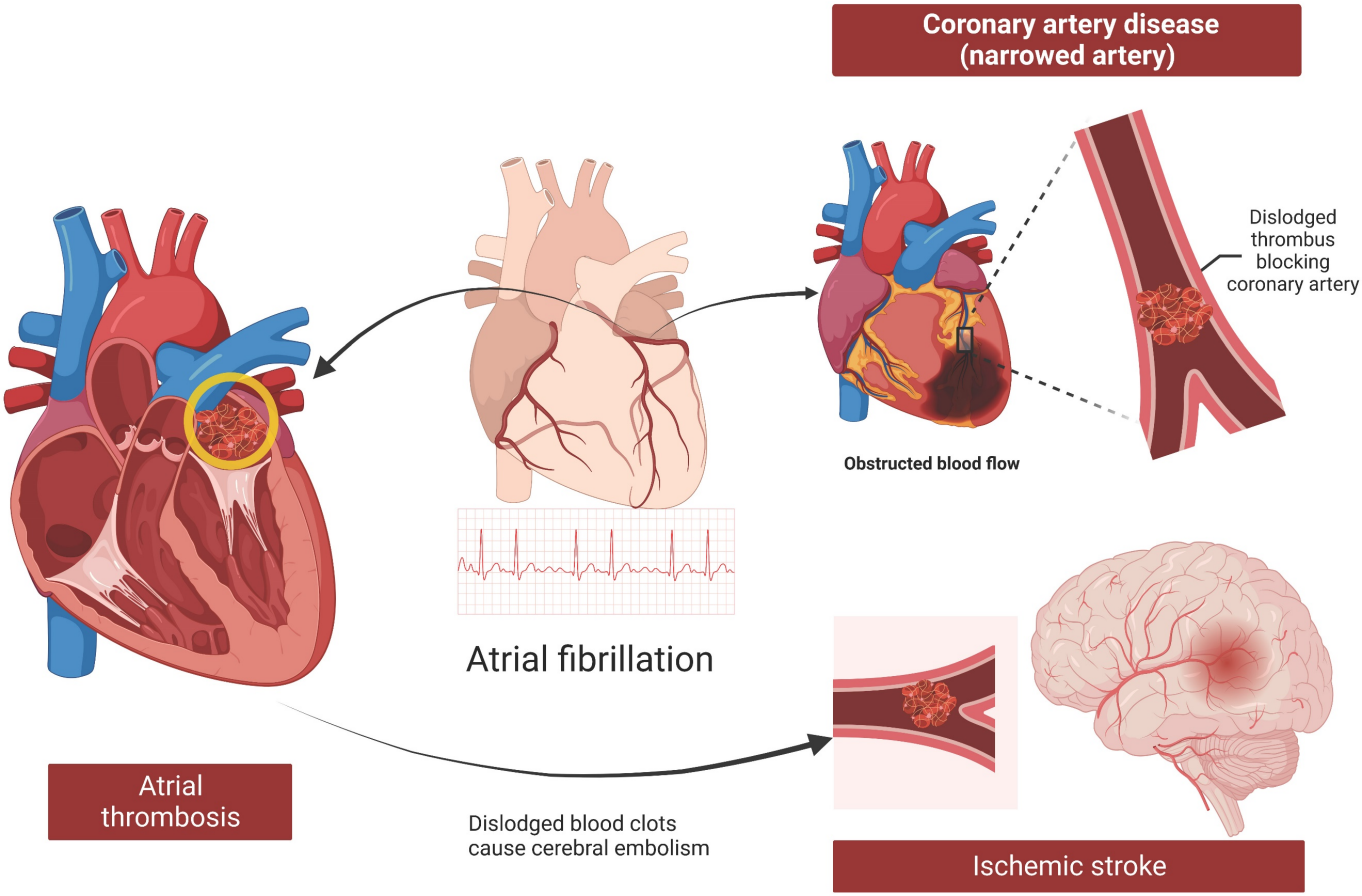
** Atherosclerosis, thrombosis, and stroke in patients with AF.** AF increases the risk of thrombosis in the left atrium due to weak atrial contractility, stagnant blood flow, and a potential prethrombotic state. Thromboembolism, where a clot formed in the heart moves to other parts of the body, is a dangerous complication of AF, with ischemic stroke being the most serious outcome. Furthermore, AF and CAD are synergistic conditions. AF can exacerbate CAD through thrombosis, inadequate blood supply, and the promotion of atherosclerosis. AF, atrial fibrillation; CAD, coronary artery disease.

Coronary artery disease (CAD) is characterized by narrowing or blockage of coronary arteries due to atheromatous plaques, leading to myocardial ischemia, hypoxia, or necrosis. CAD shares several common risk factors with AF, including smoking, obesity, hypertension, dyslipidemia, age, and race 51, 62, 63. Patients with CAD are more prone to developing AF, which can exacerbate the severity of CAD and pose greater challenges in clinical management 25 (Figure 5). Identifying common factors between these diseases can enhance prevention and treatment strategies.

Elevated Lp(a) levels are associated with an increased risk of CAD 64. Independent of CAD, Mendelian randomization studies have shown a strong association between elevated Lp(a) levels and the development of AF 18, 19. A study by Li *et al.* 65 revealed that patients with both CVD and AF were significantly older and had higher Lp(a) levels than those with CAD without AF. Multivariate logistic regression analysis revealed that increased Lp(a) levels and age were independent risk factors for AF in patients with CAD. Therefore, high Lp(a) levels may increase the incidence of AF in patients with CAD, but large-scale RCTs are needed to further validate this association.

### Association of Lp(a) with AF and ischemic stroke

AF increases the risk of thrombus formation within the left atrium due to reduced atrial contractility, stagnant blood flow, and a prethrombotic state. This predisposes patients to thromboembolism, where a clot formed in the heart can travel to other parts of the body, leading to serious complications such as ischemic stroke (Figure 5). Current evidence suggests that Lp(a) may increase the risk of ischemic stroke 66. However, the direct correlation between Lp(a) and stroke triggered by AF remains inconclusive.

Research by Aronis *et al.* 12, which focused on patients without AF, did not determine whether elevated Lp(a) levels are associated with an increased incidence of stroke in those with AF. Despite this limitation, their study revealed that high Lp(a) levels were linked to an increased risk of ischemic stroke. Arora *et al.* 67 reported that high Lp(a) levels were linked to an increased risk of ischemic stroke. However, their study did not find a sex-specific correlation between Lp(a) levels and stroke risk. Although their research suggested a potential racial disparity in Lp(a)-associated stroke risk, the results were not statistically significant, indicating that further investigation is needed. A retrospective case-control study by Fu *et al.* 68 in a Han Chinese population reached similar conclusions, noting a correlation between Lp(a) and ischemic stroke but not addressing the role of AF.

While most studies indicate a correlation between Lp(a) and ischemic stroke, few have explored whether AF plays a role in this association. This represents a significant research gap, and addressing it could lead to breakthroughs in prevention and treatment strategies for stroke and AF.

### Association of Lp(a) with AF and heart failure

Heart failure (HF) and AF frequently coexist, each exacerbating the clinical course of the other 69. Elevated Lp(a) levels have been implicated in the development and progression of HF, potentially impacting the incidence of AF in these patients. Studies indicate that Lp(a) may contribute to HF through its proatherogenic, prothrombotic, and proinflammatory properties 70-72. Elevated Lp(a) levels can lead to atherosclerosis, increasing the likelihood of ischemic events and subsequent HF. Moreover, the prothrombotic nature of Lp(a) can exacerbate HF by promoting microvascular thrombosis, further impairing cardiac function. Inflammatory responses driven by Lp(a) can also worsen HF by promoting myocardial fibrosis and adverse remodeling.

A study by Masson *et al.* 73 demonstrated a positive relationship between Lp(a) levels and HF. Among individuals with stage A or B HF, higher Lp(a) and OxPL concentrations are independent risk factors for progression to symptomatic HF or cardiovascular death 74. Given the close relationship between HF and AF, elevated Lp(a) levels may impact the development and progression of both conditions through various mechanisms. Future research should focus on investigating the specific role of Lp(a) in both HF and AF and assessing its potential as a therapeutic target.

## Therapeutic strategies for Lp(a) reduction

Among the lipoproteins associated with CVD, Lp(a) plays a crucial role in determining disease risk. Lowering Lp(a) levels can reduce the incidence of CVD and mitigate associated lipid abnormalities. Therefore, identifying effective methods to lower Lp(a) is essential for improving patient outcomes. However, current lipid-lowering drugs have had limited success in reducing Lp(a), and no specific drug for this purpose has been approved. This review summarized existing treatments for lowering Lp(a) and aimed to provide evidence for potential clinical applications (Table 1).

### Lifestyle modifications

Many cardiovascular events are associated with elevated Lp(a) levels, which are primarily genetically determined. Although lifestyle changes are key interventions for CVD, studies have shown that lifestyle modifications, such as dietary control, increased exercise, or smoking cessation, do not significantly alter Lp(a) levels. Research indicates that Lp(a) levels remain unaffected by different diets, whether they are fasted or not 75. While some studies suggest that high-carbohydrate and high-protein diets can slightly increase Lp(a) levels compared with diets rich in unsaturated fatty acids, these increases are minimal compared with the impact of alcohol consumption 76. Therefore, specific therapies targeting Lp(a) are needed to manage cardiovascular risks associated with elevated Lp(a) levels more effectively.

### Statins

Statins, which are hydroxymethylglutaryl coenzyme A reductase inhibitors, work by reducing cholesterol synthesis through competition with endogenous enzymes 77. This mechanism effectively lowers the risk of CVD. However, while statins are beneficial for managing overall cholesterol levels, they do not reduce Lp(a) levels and may even increase them 78. A meta-analysis pooling data from 45044 patients across seven studies revealed that the risk of Lp(a)-associated CVD persisted in patients on statins. The HR was 1.31 (95% CI: 1.08-1.58) before statin use and increased to 1.43 (95% CI: 1.15-1.76) after statin use, indicating an increasing trend in Lp(a) levels following statin therapy 79. Consequently, statins are not suitable for lowering Lp(a) levels.

Despite their limitations in reducing Lp(a), statins have proven effective in other cardiovascular contexts. Studies have shown that statin use is associated with a decreased risk of incident HF in a duration-dependent manner among patients with AF 80. In addition, the results from a retrospective cohort study revealed that statin therapy was independently associated with a lower incidence of stroke after adjusting for confounding variables (HR: 0.87, 95% CI: 0.78-0.97; *P* = 0.01) 81. These benefits underscore the importance of statins in specific cardiovascular conditions, even though their role in Lp(a) management remains limited.

### Lipoprotein apheresis

Patients with elevated Lp(a) levels benefit significantly from lipoprotein apheresis (LA), which has the greatest efficacy in preventing cardiovascular events. In addition to its lipid-lowering effects, LA can reduce the serum levels of proinflammatory and prothrombotic factors, decrease blood viscosity, enhance microvascular myocardial perfusion, and potentially exert beneficial effects on endothelial function 82. The German Lipoprotein Apheresis Registry summarized data on lipoprotein changes following LA treatment in 1435 patients with CVD enrolled between 2011 and 2016 83. On the basis of the results of all patients treated with LA, an average reduction of 71.1% in Lp(a) was achieved. Additionally, LA treatment was associated with a low rate of adverse events (5.9%), mostly puncture-related problems, and resulted in a 90% reduction in cardiovascular events 83. A retrospective cohort study demonstrated that after a mean duration of 7.1 years of LA treatment, patients' median Lp(a) levels decreased from 95.0 to 31.1 mg/dL (-67.3%, *P*  < 0.0001), mean LDL-C levels decreased from 1.85 to 0.76 mmol/L (-58.9%, *P*  < 0.0001), and the annual rate of major adverse cardiac events decreased from 0.34 to 0.006 (-0.33, *P*  = 0.0002) 84. These findings indicate that LA not only lowers Lp(a) and LDL-C levels but also concomitantly reduces the incidence of cardiovascular events. Moreover, Pokrovsky *et al.* 85 reported that eliminating Lp(a) from the bloodstream can reduce inflammatory and thrombotic processes within months and lead to the regression of atherosclerotic plaques within 1.5 years. In both clinical trials and real-world settings, LA treatment for 2-5 years has further demonstrated that a 60-80% reduction in Lp(a) is associated with a proportional decrease in the incidence and risk of cardiovascular events. However, no studies have investigated whether LA can reduce the incidence of AF by lowering Lp(a) levels. Nevertheless, LA remains a highly effective method for specifically reducing Lp(a). If a causal relationship between lowering Lp(a) and a reduced incidence of AF can be established, LA may provide a promising therapeutic approach for the management of AF.

### PCSK9 inhibitors

It is hypothesized that PCSK9 inhibitors reduce Lp(a) levels by increasing the number of LDL receptors, thereby increasing Lp(a) clearance. PCSK9 inhibitors not only decrease Lp(a) levels but also reduce the incidence of cardiovascular events 86. The results from the FOURIER-OLE study demonstrated that patients treated with evolocumab experienced a 23% reduction in the risk of cardiovascular death (HR: 0.77; 95% CI: 0.60-0.99; *P* = 0.04) and a 20% reduction in the risk of myocardial infarction or stroke (HR: 0.80; 95% CI: 0.68-0.93; *P* = 0.003) compared with those in the placebo group 87. A secondary analysis of the FOURIER-OLE study indicated that achieving long-term low LDL-C levels with evolocumab, as low as < 20 mg/dL (< 0.5 mmol/L), was associated with a reduced risk of cardiovascular outcomes without significant safety concerns 88. Furthermore, a meta-analysis demonstrated that PCSK9 inhibitors provide substantial and durable reductions in LDL-C levels and improve cardiovascular outcomes 89. These findings underscore the significant potential of PCSK9 inhibitors in enhancing cardiovascular health.

Two monoclonal antibodies, evolocumab and alirocumab, both of which are fully human monoclonal antibodies injected subcutaneously, have been developed and are in clinical use as PCSK9 inhibitors 46. For different baseline plasma PCSK9 levels, evolocumab resulted in a reduction in lipoprotein levels. The effect of alirocumab at the maximum dose, whether used as monotherapy or in combination, achieved a 60% decrease in plasma lipoproteins, which is consistent with the effects of evolocumab 46. From a safety perspective, the safety profile of PCSK9 inhibitors is positive, with only a few patients experiencing mild injection site reactions. However, there is some evidence that PCSK9 inhibitors do not reduce the risk of vascular wall inflammation, possibly because PCSK9 inhibitors do not significantly decrease Lp(a) in people with persistently increasing Lp(a) 90. A Mendelian randomized trial suggested that a targeted reduction in the PCSK9 concentration is significant for reducing the occurrence of AF (OR: 0.90, 95% CI: 0.83-0.97) and has a protective effect on patients at risk of AF 91. These findings suggest that a lower concentration of PCSK9 is associated with a lower risk of AF, and other methods to reduce PCSK9 concentrations may also prevent AF, providing a valid approach for treatment. However, low PCSK9 concentrations increase the risk of developing Alzheimer's disease and asthma 91.

These findings highlight the potential of PCSK9 inhibitors in reducing Lp(a) levels and associated cardiovascular events. However, despite their efficacy, PCSK9 inhibitors may not significantly impact vascular wall inflammation in patients with persistently elevated Lp(a). Furthermore, while a targeted reduction in PCSK9 concentrations shows promise in lowering the risk of AF, it is essential to consider the possible increased risks of developing Alzheimer's disease and asthma. Thus, comprehensive research and clinical evaluations are necessary to balance these benefits and risks, guiding the effective use of PCSK9 inhibitors in managing CVD.

### Olpasiran

Olpasiran, a novel small interfering RNA (siRNA) targeting PCSK9, effectively reduces PCSK9 production, leading to decreased Lp(a) synthesis in the liver. In a placebo-controlled trial, patients with ASCVD treated with Olpasiran presented significantly lower Lp(a) levels than did those receiving a placebo. The efficacy of Olpasiran in reducing Lp(a) levels is dose-dependent, with higher doses resulting in greater reductions, while adverse events remain minimal and primarily involve injection site pain 92. Consequently, Olpasiran shows promise as a therapeutic option for ASCVD patients with the aim of reducing Lp(a). Furthermore, recent results from the OCEAN(a)-DOSE study demonstrated that participants receiving doses of ≥ 75 mg every 12 weeks sustained an approximately 40-50% reduction in Lp(a) levels nearly one year after the last dose, with no significant adverse effects observed 93. Given that PCSK9 inhibitors have been shown to lower AF incidence by reducing Lp(a), it is plausible that Olpasiran may similarly reduce the risk of AF. However, further evidence is needed to confirm its therapeutic efficacy in reducing AF risk.

### Antisense oligonucleotides

Antisense oligonucleotides bind directly to messenger RNA (mRNA) in hepatocytes, inhibiting target gene protein synthesis and thereby reducing Lp(a). Like siRNA therapy, antisense oligonucleotide therapy works by binding to RNA in target cells through base pairing, inhibiting gene function. AKCEA-APO(a)-LRX is an antisense oligonucleotide that decreases Lp(a) in hepatocytes in a dose-dependent manner. Patients treated with AKCEA-APO(a)-LRX experienced an average 80% reduction in Lp(a) levels when given the highest dose 94.

Some studies suggest a relationship between small conductance Ca^2+^-activated K^+^ (SK3) channels and the development of AF, indicating that downregulating the expression of these cardiac proteins may prevent AF. In an experiment in which antisense oligonucleotides were used to knock down SK3 channels in rats, the group with downregulated SK3 protein expression presented 78% shorter episodes of AF than did the control group with artificially induced AF. There was a 68% reduction in spontaneous AF episodes, and the duration of spontaneous AF episodes was significantly shorter, with the experimental group experiencing episodes lasting 7.2 seconds compared with 29.7 seconds in the control group 95. Electrophysiological parameters were measured to verify the protective effect of SK3 channels on AF. Although the experimental group exhibited delayed prolongation of the effective refractory period, SK3 channels downregulation still conferred a protective effect against AF 95. Given the close association of SK3 channel with AF, this trial indicates significant prospects for antisense oligonucleotides in treating AF. However, this approach lacks clinical evidence, and since rats do not fully represent human electrophysiological activity, more clinical data are needed to demonstrate the effects of antisense oligonucleotides on AF.

### Cholesterol ester transfer protein inhibitors

Cholesterol ester transfer protein (CETP) inhibitors block the transfer of cholesterol esters from nonatherogenic high-density lipoprotein (HDL) particles to atherogenic lipoprotein fractions, including LDL. Anacetrapib and evacetrapib are notable CETP inhibitors, with TA-8995 emerging as a new CETP inhibitor with high tolerability. A phase 2 trial revealed that TA-8995 reduced LDL cholesterol levels by 45.3%, increased HDL cholesterol levels by 179.1%, and increased ApoA-1 levels by 63.4%. When used in combination with statins, TA-8995 further reduced LDL levels by an additional 39.8-50.2% 96. However, the impact on cardiovascular event risk requires further validation through trials.

In a Mendelian randomized evaluation trial, targeted CETP inhibition had an OR of 0.99 (95% CI: 0.96-1.01) for AF, indicating no significant association 91. Nevertheless, CETP inhibitors may reduce the incidence of CVD, including heart disease and stroke. The combined use of CETP inhibitors and PCSK9 inhibitors, which impact lipoprotein metabolism differently, could provide greater benefits. Both classes lower Lp(a) levels, and PCSK9 inhibitors have been shown to reduce the incidence of AF 91. Ongoing studies are investigating the combined effects of these inhibitors on AF, potentially providing improved prognoses for patients.

### IL-6 receptor monoclonal antibodies

Tocilizumab, a humanized monoclonal antibody, targets IL-6 receptors and is used to treat severe inflammatory conditions such as rheumatoid arthritis (RA). By inhibiting IL-6-induced Lp(a) mRNA and protein expression in human hepatocytes, tocilizumab reduces inflammation and joint damage in patients with RA 48. Studies have shown that tocilizumab can reduce Lp(a) levels in RA patients by approximately 30-40% 97. In addition, in patients with COVID-19, treatment with tocilizumab also reduces Lp(a) levels by approximately 30% 98.

IL-6 is commonly used as an indicator of inflammation and is used to gauge the efficacy of anti-inflammatory agents. Inflammation is also a known cause of AF, suggesting that IL-6 receptor monoclonal antibodies are promising for reducing AF occurrence. However, more research is needed to determine whether they reduce the incidence of AF by lowering Lp(a) levels.

## Limitations and prospects

AF, as one of the most common and persistent arrhythmias, often presents without typical clinical symptoms, making early screening, diagnosis, and treatment crucial to prevent serious adverse events. The relationship between Lp(a) and AF has shown conflicting results across different ethnic groups. For example, high Lp(a) levels are positively associated with AF in Europeans, whereas studies in Eastern populations indicate a negative association. This discrepancy highlights several limitations in current research, such as ancestral homogeneity and insufficient sample sizes, which impedes a comprehensive understanding of the global role of Lp(a).

Most current studies, primarily those utilizing Mendelian randomization and cohort analyses, are limited by a lack of ethnic diversity. To elucidate the ethnic variations in disease incidence and the impact of allele distribution, future research must include larger and more diverse populations. Furthermore, the direct connection between specific components of Lp(a) and AF remains unclear, with current findings suggesting only indirect effects through proatherogenic, prothrombogenic, and proinflammatory mechanisms. Addressing these gaps could lead to new strategies for preventing and treating AF by targeting Lp(a).

A study by Mohammadi-Shemirani *et al.* 19 using UK biobank data revealed that elevated Lp(a) levels are linked to new-onset AF, independent of ASCVD. These findings point to potential new treatments for patients with high Lp(a) levels. However, effective lipid-lowering therapies specifically targeting elevated Lp(a) levels are limited, and no approved treatments are currently available. Therefore, developing effective Lp(a)-lowering strategies is critically needed.

Emerging Lp(a)-lowering therapies, such as PCSK9 inhibitors, siRNAs, and antisense oligonucleotides, have shown promise for significantly lowering Lp(a) levels. Drugs such as Olpasiran, PCSK9 inhibitors, and TA-8995 have demonstrated minimal severe side effects in clinical trials, whereas the antisense oligonucleotide mipomersen has been associated with hepatic side effects, limiting its applicability. Additionally, nonlipid therapies addressing elevated Lp(a)-induced problems, such as antiplatelet agents, have demonstrated effectiveness in modifying coagulation and platelet aggregation 99, suggesting alternative treatment options.

Despite numerous treatment options and clinical trials showing significant reductions in Lp(a) levels, only PCSK9 inhibitors and LA have demonstrated clear improvements in clinical outcomes and reduced cardiovascular events. This finding underscores the uncertainty surrounding the optimal clinical treatment regimen for patients with elevated Lp(a). Although many drugs can lower Lp(a), their ability to reduce CVD incidence remains unclear. Reducing cardiovascular events in patients with elevated Lp(a) is essential for improving patient prognosis and quality of life. Statins and antiplatelet agents, despite not directly lowering Lp(a), may reduce cardiovascular risk through their functions. This finding requires validation in larger trials.

An important consideration is whether lowering Lp(a) in patients with elevated levels is correlated with a reduced risk of cardiovascular events. Current evidence suggests that only PCSK9 inhibitors and LA reduce both Lp(a) levels and CVD risk. These findings indicate the potential benefit of combining Lp(a)-lowering drugs with cardiovascular medications to improve patient outcomes. The ongoing HORIZON trial (trial node: NCT04023552) is expected to provide valuable insights into these relationships, significantly advancing cardiovascular event prevention.

## Conclusion

Elevated Lp(a) levels are associated with an increased risk of AF through mechanisms such as proatherogenic, prothrombotic, and proinflammatory effects. While current lipid-lowering therapies, such as statins, are ineffective at reducing Lp(a), emerging treatments, including PCSK9 inhibitors, siRNAs, and antisense oligonucleotides, show promise. LA also significantly decreases Lp(a) levels, but further study is needed to determine its impact on AF. Future research should focus on diverse populations to better understand the role of Lp(a) in AF and validate the effectiveness of new therapies.

## Figures and Tables

**Table 1:** Current therapies for Lp(a).

Therapies	Mechanism	Impact on Lp(a)	Effect on AF or CVD
Statins	Reducing cholesterol synthesis through competition with endogenous enzymes	Do not reduce Lp(a), and may even increase its level	Reduces the risk of HF and stroke events in patients with AF
LA	The blood lipids were removed by extracorporeal circulation blood purification	Lp(a) decreased by an average of 71.1% in patients treated with LA	No studies have examined using LA to lower the incidence of AF by lowering Lp(a) levels
PCSK9 inhibitors	Enhancing the number of LDL receptors	PCSK9 inhibitors not only have the clinical effect of decreasing Lp(a) but also reduce the occurrence of cardiovascular events	PCSK9 shows promise in lowering the risk of AF, but it is essential to consider the possible increased risks of developing Alzheimer's disease and asthma
Olpasiran	Effectively reduces PCSK9 production	The efficacy of Olpasiran in reducing Lp(a) levels increased with higher concentrations	No studies have examined using Olpasiran to lower the incidence of AF by lowering Lp(a) levels
Antisense oligonucleotides	Bind directly to mRNA in hepatocytes, inhibiting the target gene's protein synthesis and thereby reducing Lp(a)	Treated with antisense oligonucleotides experienced an average 80% reduction in Lp(a) levels when given the highest dose	Animal experiments have shown that antisense oligonucleotides reduce the frequency and duration of AF by downregulating SK3 channels, but clinical evidence is lacking
CETP inhibitors	Block the transfer of cholesterol esters from nonatherogenic particles to atherogenic lipoprotein fractions	TA-8995 reduced LDL cholesterol levels by 45.3%, increased HDL cholesterol levels by 179.1%, and boosted ApoA-1 levels by 63.4%	CETP inhibitors may reduce the incidence of CVD, including heart disease and stroke
IL-6 receptor monoclonal antibody	Inhibiting IL-6-induced Lp(a) mRNA	Tocilizumab can reduce Lp(a) levels in RA patients by approximately 30%-40%	No studies have examined using IL-6 receptor monoclonal antibody to lower the incidence of AF by lowering Lp(a) levels

AF, atrial fibrillation; CETP, cholesterol ester transfer protein; CVD, cardiovascular disease; HDL, high-density lipoprotein; HF, heart failure; IL-6, interleukin-6; LA, lipoprotein apheresis; LDL, low-density lipoprotein; Lp(a), lipoprotein(a); mRNA, messenger RNA; PCSK9, proprotein convertase subtilisin/kexin type 9; RA, rheumatoid arthritis; SK3, small conductance Ca^2+^-activated K^+^ channel.

## Abbreviations

AF: atrial fibrillation
Apo(a): apolipoprotein(a)
ASCVD: atherosclerotic cardiovascular disease
BMI: body mass index
CAD: coronary artery disease
CETP: cholesterol ester transfer protein
CI: confidence interval
CVD: cardiovascular disease
DBP: diastolic blood pressure
ECG: electrocardiogram
GWAS: genome-wide association studies
HDL-C: high-density lipoprotein cholesterol
HF: heart failure
HR: hazard ratio
IL-6: interleukin-6
LA: lipoprotein apheresis
LDL-C: low-density lipoprotein cholesterol
Lp(a): lipoprotein(a)
mRNA: messenger RNA
OR: odds ratio
OxPL: oxidized phospholipid
PCSK9: proprotein convertase subtilisin/kexin type 9
RA: rheumatoid arthritis
RCTs: randomized controlled trials
ROS: reactive oxygen species
SBP: systolic blood pressure
siRNA: small interfering RNA
SK3: small conductance Ca2+-activated K+ channel
